# Assessing the effects of drought stress on photosynthetic performance and physiological resistance in camphor seedling leaves

**DOI:** 10.1371/journal.pone.0313316

**Published:** 2025-01-08

**Authors:** Renjie Wang, Xingxing Qin, Huibiao Pan, Dianyun Li, Xiao Xiao, Yuke Jin, Yong Wang, Huizi Liang

**Affiliations:** 1 Guangxi Forestry Research Institute, Guangxi Key Laboratory of Special Non-wood Forest Cultivation and Utilization, Nanning, P. R. China; 2 College of Landscape Architecture and Art Design, Hunan Agricultural University, Changsha, Hunan, P. R. China; 3 Huangmian State-Owned Forest Farm in Guangxi, Liuzhou, P. R. China; Tennessee State University, UNITED STATES OF AMERICA

## Abstract

The impact of seasonal short-term drought on plant physiology and resilience is crucial for conservation and management strategies. This study investigated drought stress effects on growth, photosynthetic capacity, and physiological responses of Camphor (*Cinnamomum camphora*) seedlings in Guangxi province, China. Fertilized potted plants underwent continuous drought treatments to assess varying water supply effects. Treatments included normal water supply (CK), light drought (D1), moderate drought (D2), and severe drought (D3). Physiological indicators including net photosynthetic rate (Pn), stomatal conductance (Gs), transpiration rate (Tr), and intercellular CO_2_ concentration (Ci) were measured. Additionally, the stomatal limitation value (Ls) was calculated using the formula Ls = 1-Ci/Ca, and water use efficiency (WUE) was computed as Pn/Tr. Furthermore, parameters such as PI_ABS_ (Performance Index based on absorbed light energy), W_K_ (the ratio of variable fluorescence F_K_ at the K point to the amplitude F_O_-F_J_), V_J_ (the ratio of variable fluorescence F_J_ at the J point to the amplitude F_O_-F_P_), ΔI/I_0_ (the relative amplitude of the 820 nm light absorption curve), superoxide dismutase (SOD), peroxidase (POD), catalase (CAT), and malondialdehyde (MDA) were measured to evaluate the impact of drought stress on various physiological processes and antioxidant enzyme activities. Results showed significant decreases in base diameter growth (GD) and seedling height growth (GH) with increasing drought stress. Notably, moderate (D2) and severe (D3) drought treatments led to negative GD values. GD decreased by 23.79%, 114.85%, and 175.50% for D1, D2, and D3 treatments, respectively, while reductions of 40.00%, 73.33%, and 90.00% in GD were observed compared to the control (CK). Pn decreased significantly across treatments, with D1<CK<D2<D3 in Ci. Stomatal limit value (Ls) and water use efficiency (WUE) followed the order: D1>CK>D2>D3. Light energy transmission to PSI by the unit reaction center (REo/RC) initially increased then decreased, significantly smaller in D3 compared to D1. Conversely, heat dissipation absorbed by the unit reaction center (DIo/RC) increased notably in D3 compared to D1 and CK. PI_ABS_, W_K_, V_J_, and ΔI/I_0_ decreased over time, while Rubisco enzyme activity decreased, while proline (Pro) levels increased. Superoxide dismutase (SOD), peroxidase (POD), catalase (CAT), and malondialdehyde (MDA) levels significantly increased during D1 treatment but decreased with D2 and D3 treatments. Overall, drought severity had varying impacts on *Cinnamomum camphora* growth and photosynthetic structure, with D1 treatment maintaining normal growth and metabolic activities, while D2 and D3 treatments resulted in severe membrane damage, rendering seedlings essentially unable to survive. These findings provide a theoretical basis for implementing water management practices and conservation strategies for camphor seedlings.

## 1. Introduction

The impact of global climate change on plant growth and physiological processes is significant. With climate shifts and uneven precipitation patterns, water shortages are becoming more common, leading to decreased soil moisture [1]. Studies indicate that the water stress reduces agricultural and forestry yields more than other factors combined [2].

Guangxi, located in the East Asian tropical monsoon region, experiences variable precipitation due to the interplay of winter and summer monsoons and its complex geography. The region faces significant seasonal and inter-annual precipitation fluctuations, leading to frequent short-term deficits. The extensive karst landscape in Guangxi results in shallow soils with poor water retention, making the area particularly vulnerable to droughts [3]. This has severe implications for water resources, agriculture, and forestry [4]. Understanding tree seedlings’ growth under drought conditions is crucial for developing drought-tolerant species and effective seedling water management strategies in Guangxi, essential for successful afforestation.

The absence of soil moisture can significantly impede plants’ absorption of nutrients from the soil and reduce photosynthesis efficiency, thereby limiting overall plant growth [5]. Plant adaptation to water stress involves crucial physiological and biochemical processes, including osmotic regulation, photosynthesis, and respiration [6]. Photosynthesis is essential for plant growth and development, and its efficiency under drought conditions is a key indicator of drought tolerance [7]. Photosystem I (PSI) and Photosystem Ⅱ (PSⅡ) are critical for photosynthetic reactions and respond quickly to stressors such as drought. Techniques like rapid chlorophyll fluorescence induction and 820 nm light absorption curves provide quick, sensitive, and non-destructive assessments of the physiological conditions of these photosystems, including their light energy absorption, transmission, and dissipation [8].

In recent years, technologies have been extensively applied in research concerning changes in plant photosynthetic mechanisms under stress conditions. Studies indicate that photosynthesis is the most visibly affected physiological process by drought stress [9]. Under drought conditions, the plant’s photosynthesis rate decreases [10], along with reductions in stomatal conductance and rates of CO_2_ absorption and O_2_ release [11]. Upon exceeding a certain stress threshold, the structure and function of the photosynthetic system become inhibited [12]. Fridovich initially proposed the theory of biological free radical damage [13],suggesting that the excessive production of free radicals in plants triggers membrane lipid peroxidation, resulting in damage to the cell membrane system and, in severe cases, plant cell death. Throughout long-term evolution, plants have developed corresponding antioxidant protective enzyme systems, such as superoxide dismutase (SOD), peroxidase (POD), catalase (CAT), etc., which efficiently coordinate and remove O^2-^, H_2_O_2_, and other harmful substances. Numerous reports have demonstrated a significant relationship between protective enzyme activity and plant drought resistance [14]. Integrating growth indicators, photosystem performance indicators, and physiological resistance indicators to analyze plants’ response mechanisms to drought stress is essential for comprehensively understanding its impact on photosynthetic physiology and plant growth. This scientifically and logically sound approach holds significant practical significance for devising drought-resistant measures during the seedling stage.

Camphor (*Cinnamomum camphora*), an evergreen tree native to subtropical evergreen broad-leaved forests, is valued for its rapid growth, high-quality material, pest resistance, and pollution tolerance. It is a preferred species for afforestation and landscaping in southern China [15]. Presently, research on *Cinnamomum camphora* primarily focuses on aspects such as seedling breeding [16], medicinal value [17], and stress resistance [18]. Having been cultivated in China for over 2000 years, *Cinnamomum camphora* is now commonly planted for ornamental or shading purposes. There is also significant potential for it to be established as an important commercial forest tree due to its advantageous characteristics and versatility [19]. However, there has been limited research exploring the photosynthetic physiology and growth response of *Cinnamomum camphora* seedlings to drought environments.

Previous studies have focused on various physiological responses to drought stress, including the role of antioxidant systems, leaf water potential, and photosynthetic capacity [20]. For instance, *Cinnamomum camphora* has been shown to activate its antioxidant defense mechanisms to mitigate oxidative damage caused by drought, with a notable increase in enzymatic activity to reduce reactive oxygen species [21]. However, the scope of these studies remains limited in terms of integrating the relationship between photosynthetic efficiency, oxidative stress markers like malondialdehyde (MDA), and key enzymes such as Rubisco under prolonged drought conditions.

Our study aims to address this gap by investigating the interplay between these physiological processes in a more detailed manner. While the existing research provides a foundation, it lacks an in-depth analysis of how these factors collectively influence the plant’s overall drought tolerance and productivity. By elucidating these interactions, our research contributes to a more holistic understanding of drought resilience mechanisms in *Cinnamomum camphora* and offers insights that could inform future breeding programs focused on enhancing drought resistance. We hypothesize that *Cinnamomum camphora* seedlings will exhibit a gradient of physiological responses to increasing drought severity, with light drought (D1) treatment promoting minimal disruption to photosynthesis and growth, while moderate (D2) and severe drought (D3) treatments will lead to significant declines in photosynthetic efficiency, antioxidant enzyme activity, and overall seedling health. Specifically, we expect severe drought to cause substantial membrane damage, reducing the seedlings’ capacity for recovery and survival. The objectives of this study were: 1) To evaluate the effects of varying drought treatments (normal water supply, light drought, moderate drought, and severe drought) on base diameter growth (GD) and seedling height growth (GH) of Cinnamomum camphora seedlings. 2) To analyze the changes in key physiological indicators, including net photosynthetic rate (Pn), stomatal conductance (Gs), transpiration rate (Tr), and intercellular CO_2_ concentration (Ci), under varying drought treatments to determine the effects of water availability on photosynthetic capacity. 3) To measure the activity of antioxidant enzymes (superoxide dismutase, peroxidase, catalase) and stress indicators (malondialdehyde, proline levels) in response to different drought conditions, aiming to elucidate the physiological mechanisms of drought tolerance in *Cinnamomum camphora* seedlings.

## 2. Materials and method

### 2.1 Study site

The experiment took place from August 20 to September 5, 2021, in the germplasm resource conservation nursery of the Guangxi Zhuang Autonomous Region Forestry Science Research Institute, situated in the northern suburbs of Nanning City, Guangxi Zhuang Autonomous Region, China (N 22°56′, E 108°21′). The nursery occupies an altitude of 80~100 m. The site experiences a south subtropical monsoon climate characterized by an average annual temperature of 21.6°C. Extreme low temperatures can reach -1.5°C, while high temperatures can soar to 39.4°C. The annual rainfall averages 1350 mm, with an average relative humidity of 79% and an annual sunshine duration of 1827 hours.

### 2.2 Experimental design

The experiment employed a completely randomized design (CRD) with an indoor single-plant potting method. Each treatment involved one pot containing a single one-year-old *Cinnamomum camphora* seedling, with a total of 9 pots per treatment level. The seedlings used in the study are from a local landrace of *Cinnamomum camphora*, specifically selected for their adaptability to the environmental conditions of Guangxi. This local landrace, known for its resilience to regional stressors, was chosen due to its long history of cultivation in the area and its suitability for local afforestation and landscaping projects. Drought conditions were imposed at the nursery transplanting time after selecting healthy two-year-old *Cinnamomum camphora* seedlings that had been grown in pots for over six months. Prior to the experiment, controlled watering was conducted to achieve the specified soil moisture levels. Watering and monitoring were carried out daily at 8:00 AM and 6:00 PM using a weighing method. Throughout the treatment period, all management practices remained consistent, except for differences in soil moisture. The experimental cycle was established through preliminary testing."

The study comprised four different levels of water supply treatments based on soil water content (SWC): normal water supply (CK: SWC approximately 20%), light drought (D1: SWC approximately 15%), moderate water supply (D2: SWC approximately 10%), and severe drought (D3: SWC approximately 5%) [22]. Each treatment level was replicated 9 times (n = 9), resulting in a total of 36 (9 × 4) pots used in the experiment. The room temperature was maintained consistently at 25°C throughout the experiment to minimize external variables affecting soil moisture evaporation. The nutrient soil used for testing was red loam commonly found in local nurseries. The plastic flowerpot has an inner diameter of 35 cm and a height of 32 cm, with each pot holding 10 kg of soil. The nutrient soil had a total nitrogen content of 1.05 g/kg, total phosphorus content of 0.81 g/kg, and total potassium content of 11.13 g/kg. The soil’s field water capacity was 25%, expressed by soil volume water content (SWC), with a wilting point of 10%. To maintain the different levels of soil water content required for the treatments, natural pond water from the study area was used for soil moisture adjustments across the four treatment levels. We closely monitored and adjusted the soil water content (SWC) throughout the experiment. The specific steps for maintaining each water level treatment were as follows: Initially, the red loam soil in each pot was saturated with natural pond water to achieve a uniform moisture level, ensuring that all pots started under consistent conditions. Each treatment level (CK, D1, D2, D3) was assigned a specific SWC target, and daily measurements were taken using soil moisture sensors to monitor SWC. Watering frequencies were adjusted accordingly to maintain the target SWC for each treatment. Water was added gradually to avoid saturation, ensuring even distribution throughout the soil, while excess water was allowed to drain to prevent waterlogging. Throughout the experiment, pots were weighed regularly, and soil moisture was measured using a portable soil moisture meter to confirm that the target SWC levels were consistently met. Re-watering occurred as needed whenever the SWC dropped below the specified thresholds for each treatment.

### 2.3 Measurements

At the beginning and end of the drought treatments, the base diameter and height of all treated seedlings were measured using vernier calipers and tape measures. The net growth of base diameter (GD) and seedling height (GH) were calculated based on the observed differences over the experimental period. The formulas used are as follows: GD = D final-D initial, where D final is the final base diameter and D initial is the initial base diameter of the seedlings. GH = H final -H initial, where H final is the final height of the seedlings and H initial is the initial height.

After the treatments (15 days), three mature leaves were selected from each seedling in each treatment at the same position, and a Li-6400 portable photosynthesis instrument (Li-Cor Inc., USA) was utilized from 8:00 to 11:00 am on a sunny day to determine various physiological parameters. These parameters included the net photosynthetic rate (Pn), stomatal conductance (Gs), transpiration rate (Tr), and intercellular CO_2_ concentration (Ci) of the leaves. Additionally, the stomatal limitation value (Ls) was calculated using the formula Ls = 1-Ci/Ca. In this formula, Ls​represents the leaf stomatal conductance, Ci is the internal carbon concentration, and Ca​refers to the ambient carbon concentration. The water use efficiency (WUE) was computed as Pn/Tr. The measurements were conducted under controlled conditions, with a light intensity of 1000 μmol·m^-2^·s^-1^, CO_2_ concentration of 400 μmol·mol^-1^, leaf chamber temperature of 25°C, and leaf chamber air humidity maintained at 45%-65%. Additionally, M-PEA (Hansatech, UK) was employed to assess the chlorophyll fluorescence induction kinetic curve and 820 nm light absorption curve, directly providing relevant parameters related to photosystem performance. Parameters such as W_K_ = (F_K_-F_O_)/(F_J_-F_O_) and ΔI/I_0_ = (I_0_-I_min_)/I_0_ were calculated. The method employed for these measurements was based on the approach outlined by Strasser et al. [23] with slight modifications. Specifically, a red light with an intensity of 5000 μmol·m^-2^s^-1^ and a wavelength of 625 nm was used to induce chlorophyll fluorescence. Simultaneously, the fast chlorophyll fluorescence induction kinetics curve and the light absorption curve at 820 nm were measured. The fluorescence signal recording commenced at 10 μs and concluded at 2 s, with a total of 128 data points recorded.

After measuring photosynthesis, three leaves were collected from the same position of each seedling, pooled, wrapped in tin foil, and briefly stored in liquid nitrogen for preservation. Subsequently, they were transported back to the laboratory for the assessment of leaf physiological and biochemical indicators. The activity of ribulose 1,5-bisphosphate carboxylase (Rubisco) was determined using the UV spectrophotometer method [24], while the activity of superoxide dismutase (SOD) was measured using the nitroblue tetrazolium photochemical reduction method [25]. Additionally, peroxidase (POD) activity was assessed using the guaiacol method [26], and catalase (CAT) activity was measured through ultraviolet spectrophotometry [27]. Proline (Pro) content was quantified using the acidic ninhydrin method [28], while malondialdehyde (MDA) content was determined using the thiobarbituric acid method [29].

### 2.4 Data analysis

Statistical analyses were conducted to evaluate the effects of treatments, including normal water supply control (CK), light drought (D1), moderate water supply (D2), and severe drought (D3), on various physiological parameters such as net photosynthetic rate (Pn), stomatal conductance (Gs), transpiration rate (Tr), and intercellular CO_2_ concentration (Ci) of the leaves. Microsoft Excel 2016 was utilized for organizing the original experimental data and performing basic statistical analyses, while SigmaPlot 14.0 was employed for data visualization. SPSS 19.0 software was utilized for single-factor analysis of variance (ANOVA) and multiple comparisons using Duncan’s new multiple range method on seedling growth, photosynthetic physiological characteristics, and other indicators under different drought treatments. The significance level for all analyses was set at α = 0.05.

## 3. Results

### 3.1 Impact of drought treatment on the growth of camphor seedlings

**Fig 1:**
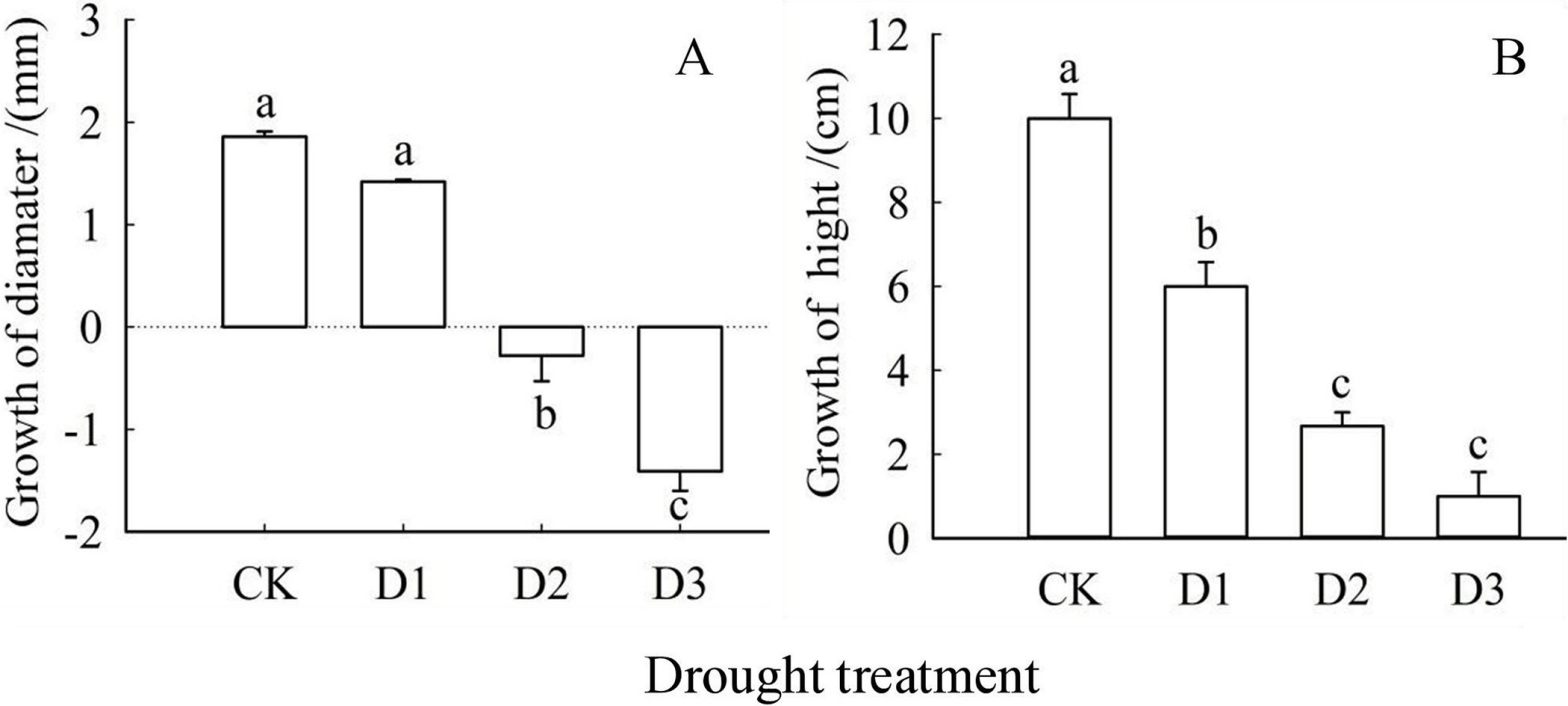
Impact of drought treatment on the growth of camphor seedlings. Note: Lowercase letters (a, b, c, d) above the bars indicate significant differences (P<0.05) among the shading treatments. The bars represent mean values with standard error (±SE) (n = 9).

**Fig 2:**
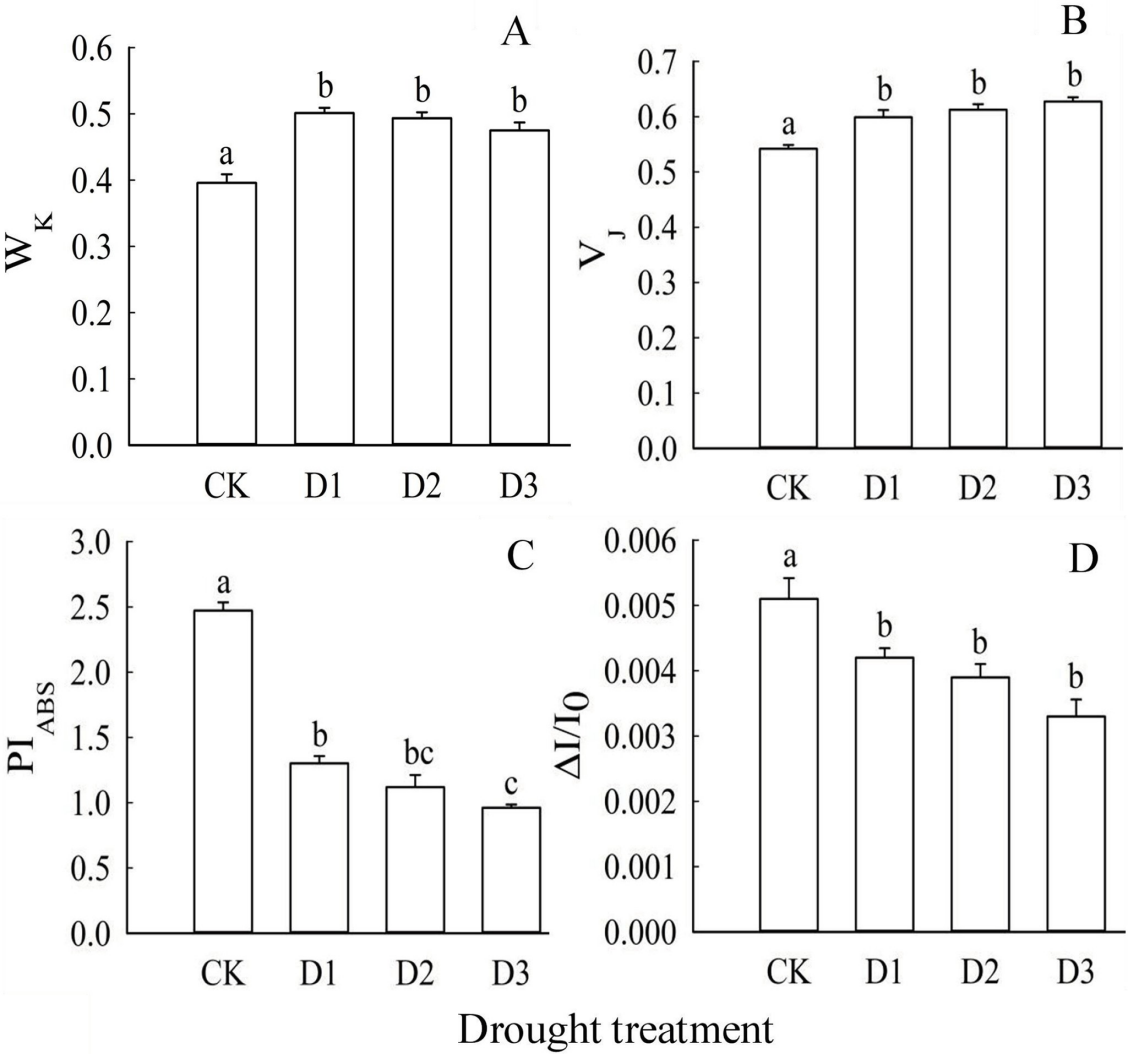
Effects of drought treatment on PSⅡ donor side/recipient side and overall PSⅡ/PS I performance of leaves. Note: Lowercase letters (a, b, c, d) above the bars indicate significant differences (P<0.05) among the shading treatments. Bars represent mean values with standard error (±SE) (n = 9).

The effect of drought treatment on the growth of *Cinnamomum camphora* seedlings is depicted in Fig 1. Both the base growth of diameter (GD) and growth of heigh (GH) of seedlings exhibited a declining trend with increasing drought severity. Significantly, both GD and GH demonstrated a significant reduction (*P*<0.05) under treatments compared to CK. A significant decrease in GD (*P*<0.05) was observed in the D2 and D3 treatments compared to the control (CK). Notably, both the moderate drought treatment (D2) and severe drought treatment (D3) resulted in negative GD values (Fig 1A). In Fig 1A, the GD decreased by 23.79%,114.85%, and 175.50% for the D1, D2 and D3 treatments, respectively, while a decreasing pattern of 40.00%,73.33% and 90.00% in GD was observed for the D1, D2, and D3 treatments, respectively compared to the CK (Fig 2B). Although there was no significant difference between D2 and D3 in GD growth, a downward trend was still observed.

### 3.2 Effect of drought treatment on photosynthetic gas exchange parameters of *Cinnamomum camphora* seedlings

It can be observed from Table 1 that as the degree of drought increases, the net photosynthetic rate (Pn) of camphor seedling leaves demonstrated a decreasing trend. Significant differences in Pn are observed between different drought treatments (*P*<0.05). The intercellular CO_2_ concentration (Ci) exhibited a trend of initially decreasing and then increasing as the degree of drought intensifies, specifically as follows: light drought treatment (D1)<control(CK)<moderate drought treatment (D2)<severe drought treatment (D3). There were significant differences in Ci between treatments (*P*<0.05). Both the stomatal limitation value (Ls) and water use efficiency (WUE) showed a trend of initially increasing and then decreasing as drought severity increased, following the pattern: D1>CK>D2>D3. Significant differences in Ls were found between different drought treatments (*P*<0.05). Additionally, significant differences in WUE were observed between CK and D1, as well as between D2 and D3 (*P*<0.05).

**Table 1:** Effect of drought treatment on photosynthetic gas exchange parameters of *Cinnamomum camphora* seedlings.

Drought Treatment	Pn/(μmol·m^-2^·s^-1^)	Ci/(μmol·mol^-1^)	Ls	WUE
Ck	7.29±0.22a	125.81±1.82a	0.70±0.01a	9.12±0.08a
D1	4.52±0.17b	63.14±14.87b	0.85±0.04b	9.33±0.79a
D2	2.18±0.24c	169.63±9.54c	0.60±0.02c	5.66±0.14b
D3	0.68±0.02d	348.68±4.59d	0.18±0.02d	1.51±0.09b

Note: Different letters indicate significant differences among the different levels of shading treatments (*P*<0.05). Data are presented as the mean ± SE (n = 9) values.

### 3.3 Effects of drought treatment on the photosystem of camphor seedling leaves

#### 3.3.1 Effect of drought treatment on leaf-specific activity parameters of *Cinnamomum camphora* seedlings

Table 2 revealed that under various drought treatments, the number of reaction centers per unit area (RC/CS_M_) in *Cinnamomum camphora* seedling leaves was significantly lower than in the control (CK) (*P*<0.05). However, no significant differences were detected between the light drought (D1), moderate drought (D2), and severe drought (D3) treatments (*P*>0.05) for this parameter. In terms of energy distribution per unit area (CS_M_), both the absorbed light energy (ABS/CS_M_) and the excitation energy used to restore Q_A_ (TR_O_/CS_M_) followed the trend: CK>D1>D3> D2, though differences between the drought treatments were not statistically significant (*P*>0.05). The light energy transferred to PSI per unit area (REo/CS_M_) showed an initial increase, followed by a decrease, with D1 being significantly lower than CK and D3 (*P*<0.05). The heat dissipation per unit area (DI_O_/CS_M_) exhibited the following trend: D3>D1>D2>CK. However, there were no significant differences between the drought treatments (*P*>0.05). When considering energy distribution per reaction center (RC), both the absorbed light energy (ABS/RC) and the excitation energy used to reduce Q_A_ (TRo/RC) mirrored the pattern observed for RC/CS_M_, being significantly lower than CK (*P*<0.05) with no significant differences among the drought treatments (*P*<0.05). As drought severity increased, the light energy transferred to PSI (REo/RC) first increased and then decreased, with D3 significantly lower than D1 (*P*<0.05). In contrast, heat dissipation per reaction center (DIo/RC) showed a continuous rise, with D3 significantly higher than both D1 and CK (*P*<0.05).

**Table 2:** Effects of drought treatment on specific activity parameters of camphor seedling leaves.

Drought Treatment	RC/CS_M_	ABS/CS_M_	TRo/CS_M_	REo/CS_M_	DIo/CS_M_
CK	30647.61	51460.33	42750.00	2114.67	8710.33
	±445.46a	±1798.78a	±1961.02a	±167.18a	±234.12a
D1	23770.03	51480.33	41572.33	2144.33	9908
	±1238.47b	±2315.15a	±1665.52a	±447.04a	±652.25a
D2	21403.35	46724.67	37107	1367.67	9617.67
	±1885.35b	±4825.05a	±3788.49a	±186.17ab	±1117.12a
D3	22653.53	48639.33	37635.33	943.00	11004
	±851.69b	±2866.74a	±2235.77a	±112.58b	±633.535a
Drought Treatment	ABS/RC	TRo/RC	REo/RC	DIo/RC	
CK	1.68±0.04a	1.39±0.04a	0.07±0.01ab	0.28±0.01a	
D1	2.17±0.03b	1.75±0.03b	0.09±0.02a	0.42±0.01b	
D2	2.18±0.04b	1.73±0.03b	0.07±0.01ab	0.45±0.02bc	
D3	2.14±0.05b	1.66±0.04b	0.04±0.01b	0.49±0.01c	

Note: Different letters indicate significant differences among the different levels of shading treatments (*P*<0.05). Data are presented as the mean ± SE (n = 9) values.

#### 3.3.2 Effect of drought treatment on energy allocation ratio and quantum yield of *Cinnamomum camphora* seedling leaves

Observations from Table 3 revealed a consistent decrease in maximum photochemical efficiency (φ_Po_) after the dark reaction as the severity of drought increases. The light drought treatment (D1), moderate drought treatment (D2), and severe drought treatment (D3) all exhibited significant reductions compared to the control (CK) (*P*<0.05). Notably, significant differences were also observed among the D1, D2, and D3 treatments (*P*<0.05). The ratio of excitons captured by the PSⅡ reaction center, which drives electron transfer downstream of Q_A_ in the electron transport chain (Ψ_o_), demonstrated a similar downward trend. Both D1, D2, and D3 treatments were significantly lower than CK (*P*<0.05), although no significant differences were found between the drought treatments themselves(*P*<0.05). Additionally, the probability of light energy captured by the PSⅡ reaction center that successfully transfers electrons to other electron acceptors in the electron transport chain beyond Q_A_ (φ_Eo_) showed a decreasing trend. In contrast, the quantum ratio of heat dissipation (φ_Do_) continued to rise, with D1, D2, and D3 treatments all significantly higher than CK (*P*<0.05). Significant differences were also noted among D1, D2, and D3 (*P*<0.05).

**Table 3:** Effects of drought treatment on leaf quantum yield or energy allocation ratio.

Drought Treatment	φ_Po_	Ψ_o_	φ_Eo_	φ_Do_
Ck	0.830±0.009a	0.458±0.007a	0.380±0.002a	0.170±0.009a
D1	0.808±0.004b	0.401±0.013b	0.324±0.009b	0.192±0.004b
D2	0.795±0.008b	0.386±0.010b	0.307±0.010bc	0.205±0.008b
D3	0.774±0.001c	0.374±0.009b	0.289±0.007c	0.226±0.001c

Note: Different letters indicate significant differences among the different levels of shading treatments (*P*<0.05). Data are presented as the mean ± SE (n = 9) values.

#### 3.3.3 Effects of drought treatment on PSⅡ donor side/recipient side and PSⅡ/PSI overall performance of camphor seedling leaves

In Fig 2, the results indicated that drought treatments significantly increased the ratio of variable fluorescence at the K point to the amplitude F_O_-F_J_ (W_K_) and the ratio of variable fluorescence at the J point to the amplitude F_O_-F_P_ (V_J_) in Cinnamomum camphora seedling leaves. This increase was evident across all levels of drought severity—light (D1), moderate (D2), and severe (D3)—with all treatments showing significantly higher values compared to the control (CK) (*P*<0.05) (Fig 2A & 2B). However, no significant differences were found among the drought treatments (D1, D2, and D3) for both W_K_ and V_J_ (Fig 2A & 2B). (Fig 2A & 2B). Additionally, the performance index based on absorbed light energy (PI_ABS_) and the relative amplitude of the 820 nm light absorption curve (ΔI/I_0_) exhibited a consistent decreasing trend. PI_ABS_, which reflects the overall performance of PSⅡ, was significantly lower in D1, D2, and D3 treatments compared to CK (*P*<0.05), with a notable difference between D3 and D1 (*P*<0.05) (Fig 2C). Similarly, ΔI/I_0_, which assesses the maximum redox activity of PS I, was significantly reduced in D1, D2, and D3 treatments compared to CK (*P*<0.05) (Fig 2D). There were no significant differences among treatments D1, D2, and levels of drought severity—light (D1), moderate (D2), and severe (D3)—with all treatments showing significantly higher values compared to the control (CK) (*P*<0.05) (Fig 2A & 2B). However, no significant differences were found among the drought treatments (D1, D2, and D3) for both W_K_ and V_J_ (Fig 2A & 2B). (Fig 2A & 2B). Additionally, the performance index based on absorbed light energy (PI_ABS_) and the relative amplitude of the 820 nm light absorption curve (ΔI/I_0_) exhibited a consistent decreasing trend. PI_ABS_, which reflects the overall performance of PSⅡ, was significantly lower in D1, D2, and D3 treatments compared to CK (*P*<0.05), with a notable difference between D3 and D1 (*P*<0.05) (Fig 2C). Similarly, ΔI/I_0_, which assesses the maximum redox activity of PS I, was significantly reduced in D1, D2, and D3 treatments compared to CK (*P*<0.05) (Fig 2D). There were no significant differences among treatments D1, D2, and D3 in ΔI/I_0_.

### 3.4 Effects of drought treatment on the activities of dark reaction enzymes and antioxidant enzymes in the photosystem of camphor seedling leaves

**Fig 3:**
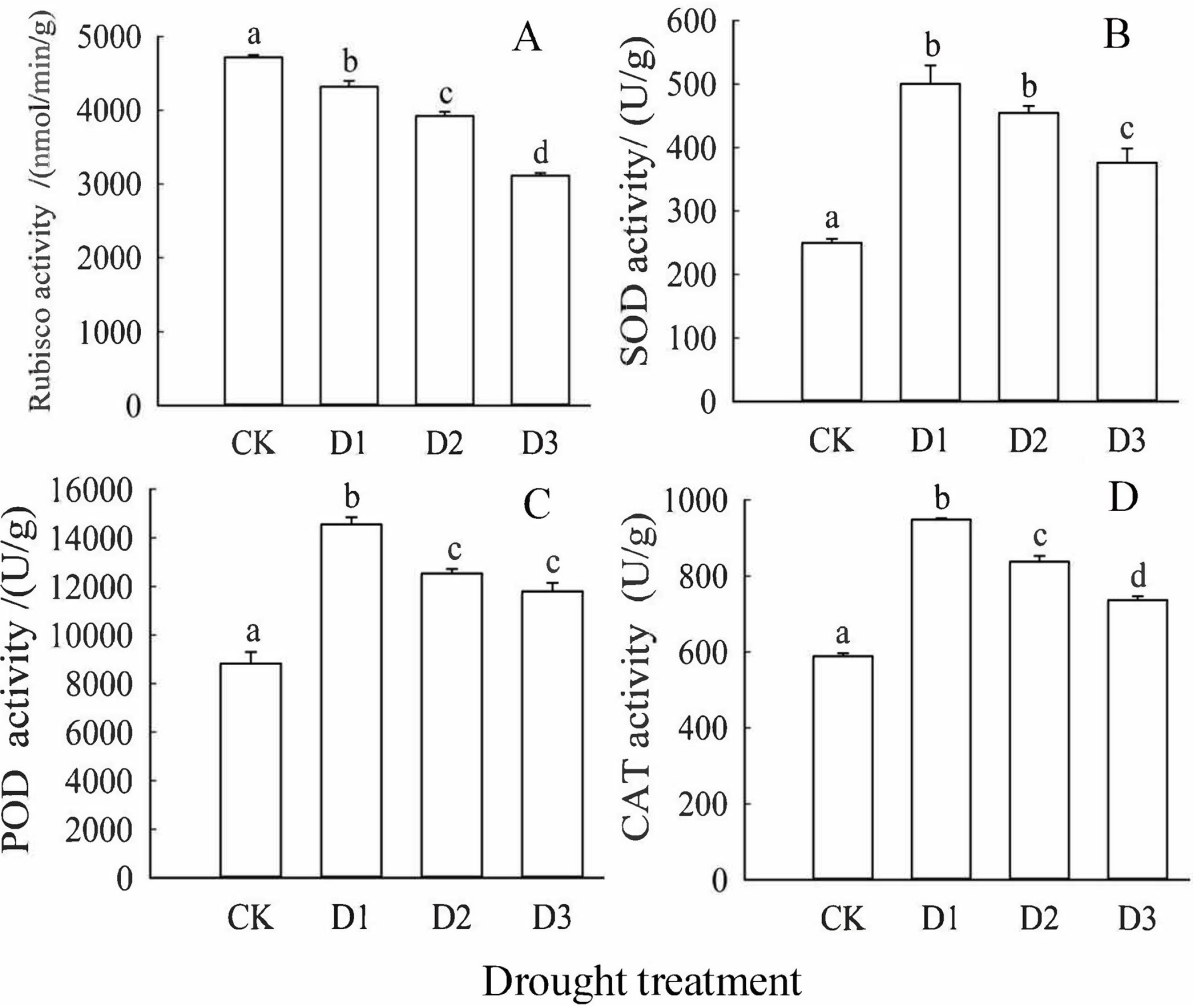
Effects of drought treatment on the activities of dark reaction enzymes and antioxidant enzymes in the photosystem of camphor seedling leaves. Note: Lowercase letters (a, b, c, d) above the bars indicate significant differences (*P*<0.05) among the shading treatments. Bars represent mean values with standard error (±SE) (n = 9).

Fig 3 illustrates that as drought treatment intensity increases, the activity of 1,5-bisphosphate ribulose carboxylase (Rubisco) in the leaves of *Cinnamomum camphora* seedlings demonstrates a clear declining trend, following the order of CK>D1>D2>D3 (*P*<0.05) (Fig 3A). Significant differences were also noted among the CK, D1, D2, and D3 treatments (*P*<0.05). In contrast, the activity of superoxide dismutase (SOD) initially increased and subsequently decreased, exhibiting the following pattern: D1>D2>D3>CK (Fig 3B). Notably, D1, D2, and D3 treatments all showed significantly higher SOD activity compared to CK (*P*<0.05), with D3 being significantly lower than both D1 and D2 (*P*<0.05). Moreover, drought treatments consistently enhanced peroxidase activity (POD), with significant differences observed among the CK, D1, D2, and D3 treatments (*P*<0.05). The trend for POD revealed an initial increase followed by a decrease, ordered as D2>D1>D3>CK (Fig 3C). Similarly, catalase activity (CAT) significantly increased under drought treatments (*P*<0.05), with the activity pattern reflecting an initial rise followed by a decline: D1>D2>D3>CK. Significant differences were found among the CK, D1, D2, and D3 treatments (*P*<0.05), with D3 showing significant differences compared to both D1 and D2 (*P*<0.05) (Fig 3D).

### 3.5 Effects of drought treatment on osmotic regulatory substances (Proline) and membrane lipid peroxidation products (MDA) in camphor seedling leaves

**Fig 4:**
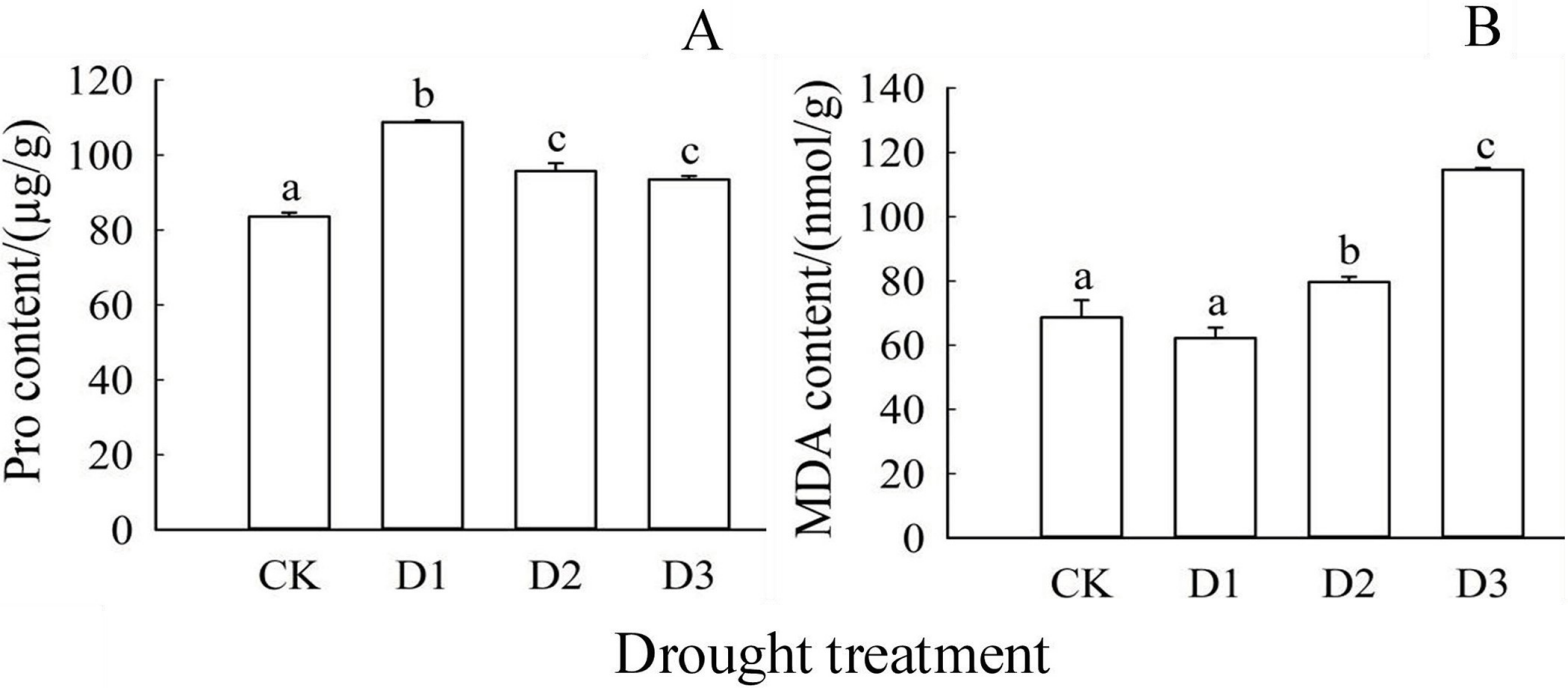
Effects of drought treatment on osmotic regulatory substances (Proline) and membrane lipid peroxidation products (MDA) in camphor seedling leaves. Note: Lowercase letters (a, b, c, d) above the bars indicate significant differences (*P*<0.05) among the shading treatments. Bars represent mean values with standard error (±SE) (n = 9).

From Fig 4, it was evident that as the drought treatments increased, the level of proline (Pro) in camphor seedling leaves initially slightly increased at D1 and D2, then increased again at D3 treatment, showing a pattern of D3>D1 = D2>CK (Fig 4A). These differences were statistically significant (*P*<0.05), with all drought treatments (D1, D2, and D3) displaying significantly higher Pro levels compared to the control (CK) (*P*<0.05). Notably, the severe drought treatment (D3) exhibited the highest Pro concentration, followed by the light drought (D1) and moderate drought (D2) treatments, while CK recorded the lowest Pro level. In contrast, malondialdehyde (MDA) concentration, an indicator of membrane lipid peroxidation, significantly increased with escalating drought severity, beginning from the D2 treatment and reaching its peak at D3. The trend for MDA levels was D3>D2>D1 = CK (Fig 4B), with significant differences noted among the D3, D2, and D1 treatments (*P*<0.05).

## 4. Discussion

Drought stress is widely recognized for its detrimental effects on plant growth and photosynthesis, often leading to symptoms such as wilting, leaf shedding, and, in extreme cases, plant death [30]. The findings of this study indicated that increasing drought stress significantly impairs the growth of *Cinnamomum camphora* seedlings, particularly in terms of base diameter growth (GD) and seedling height growth (GH). Under moderate drought treatment (D2) and severe drought treatment (D3), GD values consistently remained negative, suggesting that prolonged drought or severe soil moisture deficiency can lead to excessive water loss, resulting in stunted growth and stem shrinkage, which is consistent with previous research results [31]. Both stomatal and non-stomatal limitations are critical indicators of damage to plant photosynthetic capacity [32]. In this study, the photosynthetic capacity of *Cinnamomum camphora* leaves was primarily constrained by stomatal factors, with less impact observed on the photosystem and dark reaction processes [33]. A decrease in intercellular CO₂ concentration (Ci) accompanied by an increase in the stomatal limitation value (Ls) suggests that the reduction in net photosynthetic rate (Pn) is predominantly due to stomatal limitations. Conversely, an increase in Ci coupled with a decrease in Ls indicates that the decline in Pn is mainly driven by non-stomatal limitations [34]. Compared to the control (CK), the net photosynthetic rate decreased under light drought treatment (D1), accompanied by an increase in Ci and a decrease in Ls. In contrast, under moderate (D2) and severe (D3) drought conditions, both Pn and Ci decreased. These findings imply that the reduction in photosynthetic carbon assimilation capacity during the D1 treatment is primarily due to stomatal limitations, while non-stomatal limitations become more significant under D2 and D3 conditions. Research has demonstrated that plants can enhance their resistance to drought stress by increasing water use efficiency (WUE) [35]. However, as drought stress intensifies, the physiological balance within plant leaves is disrupted, leading to significant reductions in all functional aspects, including WUE [36]. In this study, WUE of *Cinnamomum camphora* leaves exhibited a slight increase under light drought (D1) treatment but experienced significant decreases under moderate (D2) and severe (D3) drought treatments. This suggests that the leaves of *Cinnamomum camphora* were under considerable duress during severe drought conditions. Previous studies have shown that *Cinnamomum camphora* exhibits significant reductions in stomatal conductance and transpiration under drought stress, which helps conserve water but can also limit photosynthetic activity [37]. Additionally, increases in osmolyte accumulation, such as proline, help maintain cell turgor during drought, though oxidative stress markers like malondialdehyde (MDA) also rise, indicating increased oxidative damage [38]. Furthermore, hormonal signaling pathways, particularly those involving abscisic acid (ABA), play a critical role in drought tolerance, influencing stomatal closure and stress response mechanisms, though the downstream effects of ABA on photosynthetic enzyme activity require further exploration [39].

The observed changes in the number of reaction centers per unit leaf area (RC/CS_M_) indicate that varying degrees of drought treatment have led to the inactivation or cleavage of certain reaction centers within the leaves [40]. Notably, while drought treatment did not significantly affect the light energy absorbed per unit area (ABS/CS_M_), it did have a substantial impact on the light energy captured per unit area (TR_O_/CS_M_) in the camphor leaves examined in our study. This phenomenon can be attributed to the plant’s adaptive responses to water stress. Our findings suggest that *Cinnamomum camphora* retains its capacity to absorb and utilize light energy even under drought conditions, highlighting its resilience to environmental challenges associated with photosynthesis. Furthermore, under different levels of drought stress (D1, D2, D3), the light energy absorbed per active reaction center (ABS/RC) increased, possibly serving as a compensatory mechanism for the reduced water availability [41]. Furthermore, the excitation energy (TRo/RC) and heat dissipation (DIo/RC) of the primary quinone acceptor (Q_A_) increased, signifying enhanced energy consumption efficiency among the active reaction centers [42]. Notably, no significant differences were observed in the processes of light energy absorption and capture across the various drought treatments (D1, D2, D3), suggesting that drought stress had a minimal impact on these processes in camphor leaves. This resilience highlights the plant’s ability to sustain essential photosynthetic functions even in challenging conditions. However, the transfer of light energy to PSI per unit area (REo/CS_M_) and per unit reaction center (REo/RC) exhibited a slight increase during the D1 treatment, followed by a decrease in the D2 treatment, with the most substantial decline observed during the D3 treatment. This trend indicates an imbalance in electron transfer between the photosystems under severe drought conditions Consistent with previous findings [43], our study demonstrated that as drought severity increased, the net photosynthetic rate (Pn) decreased, φ_Po_ significantly declined, and φ_Do_ increased significantly. Moreover, the quantum yield of light energy absorbed by the reaction center for electron transfer (φ_Eo_) continued to decrease in our study, ultimately impeding photosynthesis. This was because the rise of the K point in the fast chlorophyll fluorescence OJIP curve has been widely accepted as an indicator of the degree of injury to the PSⅡ donor side (OEC), and W_K_ was used to represent the change of the K point. An increase in W_K_ indicates damage to the PSⅡ donor side. Additionally, the J point reflected the degree of closure of the active reaction center at 2 ms under illumination, and an increase in V_J_ indicates injury to the PSⅡ receptor side [44]. The results of this study demonstrated that W_K_ and V_J_ under different degrees of drought treatments were greater than those of the control (CK). However, the changes between different drought treatments were not significant, indicating that under water drought pressure, all degree of drought damaged to both the photosystem Ⅱ (PS Ⅱ) donor side and acceptor side was significantly higher than that of the control. Nevertheless, as the degree of drought increased, the degree of injury to both the PSⅡ donor side and acceptor side did not exhibit a significant increase. Additionally, the parameter PI_ABS_, providing a comprehensive assessment of PSⅡ’s response to environmental factors [45], significantly decreased as drought severity increased, indicating a decline in PSⅡ activity. The impairment of PSI performance under different drought treatments was consistent across treatments, suggesting that PSI performance was compromised by drought stress, and the degree of damage was consistent across different treatments. The performance of PSI was influenced by the electron transfer capacity of upstream PSⅡ and the activity of the downstream carboxylation system [46]. In this study, as the performance of PSⅡ continued to decline and the quantum yield of light energy absorbed by the reaction center for electron transfer decreased, the damage to PSI was slowed down. However, the performance of PSI was closely linked to the enzyme activity of the downstream carboxylation system. This can be explained by the fact that a decrease in the activity of the carboxylation system’s enzyme will lead to the accumulation of photosynthetic reducing power NADPH, resulting in the excess accumulation of PSI electrons and a decline in its performance [47].

The activity of Rubisco, a key enzyme for carbon assimilation, significantly decreased in our study, leading to reduced carbon assimilation ability. Overall, our findings, supported by existing literature, highlight the detrimental impact of drought stress on photosynthetic processes in camphor leaves, with implications for carbon fixation and plant productivity [48]. Research has consistently highlighted the pivotal role of antioxidant enzymes such as superoxide dismutase (SOD), peroxidase (POD), and catalase (CAT) in plants’ defense against oxidative stress [49]. These enzymes are essential for maintaining redox balance and protecting plants from free radical damage. Under moderate drought stress, the activities of SOD, POD, and CAT increase progressively, aiding in sustaining normal plant growth and metabolic functions. However, when drought stress surpasses the plant’s tolerance threshold, it leads to a decline in the activities of these enzymes, exacerbating internal environmental damage [50]. The present study observed a significant rise in the activities of SOD, POD, and CAT during the D1 treatment, suggesting that it enabled plants to regulate themselves and maintain normal functions. Conversely, under D2 and D3 treatments, there was a notable decrease in these enzyme activities, indicating severe internal damage to camphor leaves.

Proline (Pro) serves as a vital organic solute osmotic regulator, crucial for maintaining cell osmotic pressure and stabilizing cell structure and function during environmental stress [51]. The study revealed that both D1 and D2 treatments led to similar increases in Pro levels compared to the control, while D3 treatment exhibited significantly higher levels, indicating an intensified impact on camphor leaf cell osmotic pressure. This likely underscores the severity of D3 treatment compared to the other two Malondialdehyde (MDA) content is an important indicator of membrane lipid peroxidation, reflecting the extent of plant stress [52]. The study found that MDA content initially decreased and then increased, reaching its lowest point under D1 treatment, suggesting minimal membrane damage. Conversely, under D2 and D3 treatments, MDA levels increased, indicating heightened membrane lipid peroxidation and increased damage due to excessive drought stress.

The outcomes of our study provide valuable insights into the physiological and biochemical responses of *Cinnamomum camphora* seedlings to drought stress. By identifying specific drought-tolerant traits and understanding their underlying mechanisms, breeders can focus on developing and selecting varieties with enhanced drought resistance. Key traits, such as higher Rubisco activity and lower malondialdehyde (MDA) levels, are associated with improved photosynthetic performance and reduced oxidative damage under drought conditions [53]. This knowledge enables breeders to design targeted breeding programs aimed at improving drought tolerance, leading to the development of more resilient varieties that can maintain productivity in adverse conditions [54]. These advancements are crucial for sustaining crop yields in the face of increasing climate variability. From a Sustainable Development Goals (SDG) perspective, the outcomes of our study are significant for promoting sustainable agricultural practices. By identifying drought-tolerant traits and understanding their physiological basis, farmers can make informed decisions about crop management and selection. This can lead to improved resilience of agroforestry systems to climate variability, supporting food security and sustainable land management practices.

## 5. Conclusions

In our study, we observed that increasing drought severity adversely affected the growth However, under moderate (D2) and severe drought treatments (D3), the photosynthetic capacity of camphor leaves was predominantly constrained by non-stomatal factors, leading to significant impairment of both the photosystem and dark reaction processes. This resulted in stem shrinkage and nearly inhibited growth. With increasing drought severity, both photosystem Ⅱ (PSⅡ) and photosystem I (PSI) experienced varying degrees of damage, which further reduced carbon assimilation capacity. Specifically, PSⅡ performance in camphor leaves declined significantly across different drought treatments, while the degree of damage to PSI remained consistent. Moreover, as drought severity intensified, the probability of absorbed light energy transferring electrons to acceptors beyond Q_A_ in the electron transport chain (φ_Eo_) continued to decrease, ultimately diminishing the energy ratio available for carbon fixation. *Cinnamomum camphora* responded to increased drought severity by moderating leaf damage through adjustments in physiological resistance indicators, despite ongoing damage to the membrane system.

During the D1 treatment, the activities of superoxide dismutase (SOD), peroxidase (POD), and catalase (CAT) increased, with CAT showing the most significant rise, which helped camphor leaves effectively resist drought damage and maintain normal plant growth and metabolism. In contrast, under D2 and D3 treatments, drought severely compromised the internal environment of camphor leaves, leading to reduced activities of SOD, POD, and CAT, and resulting in substantial damage to the membrane system, which ultimately hindered the survival of camphor seedlings. These findings provide valuable scientific insights that can inform the development of optimal irrigation schedules and water conservation strategies to enhance the growth and survival rates of *Cinnamomum camphora* under drought conditions.

## Supporting information

S1 Data (XLS)

S2 Data (XLS)

S3 Data (XLS)

S4 Data (XLS)

S5 Data (XLS)

S6 Data (XLS)

S7 Data (XLS)
